# Development and validation of a Sarcopenia Geriatric Scale (SARCO-GS): a new short scale for the screening of sarcopenia

**DOI:** 10.3389/fendo.2023.1192236

**Published:** 2023-08-10

**Authors:** Oscar Rosas-Carrasco, Isabel Omaña-Guzmán, Ana Isabel García-González, Armando Luna-López

**Affiliations:** ^1^ Geriatric Assessment Center, Health Department, Iberoamerican University, Mexico City, Mexico; ^2^ Pediatric Obesity Clinic and Wellness Unit, Hospital General de México “Dr. Eduardo Liceaga”, Mexico City, Mexico; ^3^ Rehabilitation Medicine Service, Hospital General de México “Dr. Eduardo Liceaga”, Mexico City, Mexico; ^4^ Departamento de Investigación Básica, Dirección de Investigación, Instituto Nacional de Geriatría, Mexico City, Mexico

**Keywords:** sarcopenia, scale, validation, screening, SARCO-GS, validity

## Abstract

**Introduction:**

Sarcopenia is a highly prevalent disease associated with adverse outcomes such as falls, disability, and death. The current international consensuses agree that muscle strength, muscle mass, and gait speed must be included in the definition. However, these proposed criteria require objective measurements that are not available for most populations. Since the timely identification of sarcopenia is a priority, several subjective screening scales have been developed; however, they have some limitations due to their low sensitivity. The objective of this work was to develop and validate SARCO-GS, a new short scale to screen sarcopenia that is affordable, easy, and accessible for all clinical care settings.

**Methods and materials:**

The development of the SARCO-GS included four stages: (1) Review and analysis of documentary sources, (2) Contextualization of the theoretical model of sarcopenia, (3) Scale conformation, and (4) Reliability and validity analyses. SARCO-GS was validated in the FraDySMex study, which is a longitudinal cohort of community-dwelling adults.

**Results:**

In the studied population (n=852), the average age was 68.9 years (SD 10.21) and 80.1% of the participants were women. SARCO-GS is a seven-item scale with an innovative structure that included five subjective questions (gait speed, muscular strength, muscle mass) and two measurements of muscular strength and muscle mass (Chair stand test and calf circumference). The results regarding criterion validity showed that the cut-off point ≥ 3 had good sensitivity (77.68%) versus the EWGSOP2 consensus, with an adequate Area Under the Receiver Operating Characteristic (AUC) (0.73), in addition to showing higher values of sensitivity and AUC than SARC-F and SARC-CalF using as reference the same consensus. Furthermore, SARCO-GS presented good predictive validity for functional dependence (HR=2.22, p=0.046) and acceptable correlation with other related measurements (construct validity). Regarding reliability, the scale showed acceptable internal reliability (correlation between items and total score: 0.50 to 0.70). After the validation analysis, the scale was adapted to English.

**Conclusions:**

The SARCO-GS is a novel scale to screen sarcopenia with high sensitivity, good construct, predictive validity, and internal reliability that may be useful for health professionals in different clinical settings and for clinical research.

## 1 Introduction

Sarcopenia is associated with age, and it is a common condition among older adults; however, most cases of sarcopenia are undiagnosed (1). A recent meta-analysis (2) estimated that the global prevalence of sarcopenia in older adults (≥60 years) ranged from 10% to 27% depending on the diagnostic criteria used for the evaluation. The main factors related to sarcopenia development are aging, low physical activity, some diseases (i.e., bone and joint diseases, and endocrine and neurological diseases), as well as nutritional factors such as an inadequate intake of energy, macronutrients, and micronutrients. These factors cause a set of alterations in skeletal muscle homeostasis, including mitochondrial dysfunction, neural plaque changes, motor neuron loss, oxidative stress, inflammation, and changes in hormones and growth factors, which are responsible for the loss of muscle mass and muscular strength (1). Several studies have identified that sarcopenia increases the risk of mortality (3), cognitive impairment (4), cardiovascular diseases (5), and functional disability (6), among other adverse health outcomes (6). There is no consensus about the clinical definition of sarcopenia (1, 7, 8). However, the existing definitions agree that it is a skeletal muscle disorder characterized by a loss of quantity and quality of muscle and low muscular strength (1, 7–10). In addition, the European Working Group on Sarcopenia in Older People (EWGSOP2) (10) and the Asian Working Group for Sarcopenia (AWGS) (9) included the presence of low physical performance (gait speed or chair stand). The EWGSOP2 (10), the AWGS (9), and the Foundation for the National Institutes of Health (FNIH) (11) have developed consensuses to diagnose sarcopenia based on objective measurements. These consensuses consider cut-off points for muscular mass, measured by dual-energy X-ray absorptiometry (DXA) or bioelectrical impedance analysis (BIA), and muscular strength measured by a manual dynamometer to establish the diagnosis of sarcopenia. The main limitation of these criteria is that they require expensive equipment to perform the measurements, making them unaffordable in clinical settings and communities. Besides, the different criteria for diagnosis reflect a need for more consensus regarding the cut-off points. However, there is a general agreement on the urgency of the call for action regarding early identification through screening scales and tests and the treatment of sarcopenia (8, 10, 11).

Given all the above, validated subjective screening scales have been developed comprising the self-report of factors related to muscle mass and muscle strength (12). The most used scale is the SARC-F (13), which has shown excellent predictive validity for adverse outcomes but low sensitivity (from 28.9 to 55.3% versus EWGSOP2); this is reported in several studies and summarized in a systematic review and meta-analysis which concluded that this scale is not optimal for sarcopenia screening (14). Subsequently, the SARC-CalF scale (15) was developed with the aim of improving sensitivity by including the measurement of calf circumference; this adjustment has shown to have a better sensitivity (from 33% to 66%) (15). However, another limitation of SARC-F and SARC-CalF is the inclusion of the number of falls within the factors evaluated to perform the diagnosis. Falls are a medium- and long-term adverse consequence of sarcopenia; therefore, this affects the early identification of individuals at risk to present adverse outcomes. Although other scales like the Mini Sarcopenia Risk Assessment (MSRA) questionnaire (16) and SarSA-Mod (17) present good or excellent sensitivity, they are focused on evaluating characteristics related to the risk of sarcopenia and not specifically to its presence.

Considering the above, our objective was to develop and validate SARCO-GS, a new short scale for the screening of sarcopenia that is affordable and easy to use, with good sensitivity, and accessible for all clinical care settings.

## 2 Methods and materials

### 2.1 Study design and population

The present study includes a longitudinal and cross-sectional data analysis on individuals aged 50 years or older participating in the FraDySMex study (Frailty, Dynapenia, and Sarcopenia in Mexican Adults). The details of the FraDySMex study (design and selection of participants) are available in other publications (18). In brief, it is a cohort study (panel study) of community-dwelling adults, mainly from three municipalities in the southeast of Mexico City. The inclusion criteria for the present study were (1): individuals who were able to move with or without assistive devices, (2) individuals who were able to answer the study questionnaire for themselves or with the help of a caregiver, (3) individuals who scored 10 points or fewer in the Mini-Mental State Examination (MMSE), and (4) individuals whose objective and subjective measurements were completed. The exclusion criteria were: (1) individuals who were institutionalized; (2) individuals with decreased alertness; and (3) the presence of any acute or chronic condition that, according to the opinion of the medical staff, could affect the individual’s ability to answer the proposed questionnaire and complete the objective evaluation. The study had a three-round design: the first round was carried out in 2014 (n=282), the second round in 2015 (n=457), and the third round in 2019 (n=852). In all rounds, the individuals underwent a series of objective and subjective evaluations by a multidisciplinary team at the Geriatric Assessment Center at the Ibero-American University and the Functional Evaluation Research Laboratory at the National Geriatric Institute in Mexico City. This study was approved by the Ethics Committee of the Angeles Mocel General Hospital and registered by the National Institute of Geriatrics (DI-PI-002/2014) and by the National Bioethics Commission (CONBIOETICA-09-cei-013- 20170517/2019). The informed written consent of all individuals was obtained.

### 2.2 Measurements

#### 2.2.1 Sarcopenia

The diagnosis of sarcopenia was made using the EWGSOP2, FNIH, and AWGS consensuses, and the following measurements were considered:

1. Muscle mass: The body composition was measured by dual-energy x-ray absorptiometry (DXA) (Hologic Discovery-WI; Hologic, Bedford, MA). For the EWGSOP2 (10), the appendicular skeletal muscle mass (ASM) was calculated as the sum of the appendicular lean mass minus the bone mineral content of both arms and legs; the cut-off point for this measurement was <20 kg for men and <15 kg for women. For the FNIH consensus (11), the ASM/Body mass index (BMI) was used (cut-off points: <0.798 for men and <0.512 for women). Whereas for the AWGS (9), the skeletal muscle mass index (SMI) was obtained by dividing the ASM by the squared height (cut-off points: <7.00 kg/m^2^ for men and <5.40 kg/m^2^ for women).

2. Muscle strength: Grip strength was measured with a hydraulic hand dynamometer (Jamar, Duluth, MN). Three measurements were taken from each hand, and the highest result for the dominant hand was considered the final value. The cut-off points for the EWGSOP2 (10) were <27 kg for men and <16 kg for women; for the FNIH (11), they were <26 kg for men and <16 kg for women, while for the AWGS (9) they were <28 kg for men and <18 kg for women.

#### 2.2.2 Other variables

- Data on age (50-69, 70 years and older), sex (male, female), and marital status (married/consensual union, single/divorced, widow/widower) were obtained from the questionnaires applied in each evaluation round.

- Anthropometric measurements. Weight was measured with a Body Composition Analyzer (Seca MBCA514), height was measured with a stadiometer (Seca 264), and the Body Mass Index (BMI) was estimated by dividing the weight by the squared height. The calf circumference was measured three times with an anthropometric tape (Seca 201). The first measurement was considered for the analysis since there were no significant differences between the three measurements. The cut-off point to screen low muscle mass was the one proposed in the SARC-CalF (≤33 cm for women and ≤34 cm for men) (15).

- Chair stand test. This test consisted of sit-to-stand repetitions (five times) while measuring the time it took the individual to execute the action; we considered this as an indicator of muscular strength. The considered cut-off point was the one proposed by the EWGSOP2 (>15 seconds).

- Quality of life. This was assessed by employing the visual analog scale from the EuroQol (EQ-VAS) (from 0 to 100 total score) (19).

- Functional dependence. This was evaluated by observing the ability to perform instrumental activities of daily living (IADL) with the Lawton Instrumental Activities of Daily Living Scale (Functional disabilit**y** ≤7 points) (20), while the basic activities of daily living (ADL) were assessed with the Barthel Index (from 0 to 100 total score) (21).

- Comorbidity. This was measured with the Charlson comorbidity index (22) (low comorbidity: 0-2 points, high comorbidity ≥3 points).

- Nutritional status. This factor was evaluated with the Mini Nutritional Assessment (MNA) (from 0 to 21 total score) (23).

- Gait speed. This was measured with the GAITRite G walk System (m/seg).

- Physical performance. This was evaluated by employing the Short Physical Performance Battery (SPPB) (from 0 to 12 total score) (24).

- Phase angle. This was evaluated with a bioelectrical impedance tetrapolar, brand SECA-mBCA 514, at a frequency of 50Hz (from 0 to ∞ total score in grades).

- Cognitive impairment. This was assessed by the MMSE (score ≤23 in the case when the years of study were ≥5; score ≤19 if the years of study were between 1 and 4; score ≤ 16 if the years of study were <1) (25).

- Depression symptoms. They were evaluated with the seven-item Center for Epidemiologic Studies depression scale short form (CESD-7) (≥5 points) (26).

### 2.3 Development of SARCO-GS

The development of the SARCO-GS included three stages (1): a literature review about scales and consensuses to evaluate sarcopenia in older adults, (2) contextualization of the theoretical model, and (3) conformation of the scale.

#### 2.3.1 First stage: review and analysis of documentary sources

This stage included a review of the scientific literature in the PubMed electronic database. The search strategy was carried out using the following Medical Subject Heading (MESH) terms: “Sarcopenia” AND “Diagnosis” AND “Aging” AND “Consensus” OR “Validation Study”. In addition, the employed keywords were development, validation and scale, tool or test or instrument or screening or index or battery. The inclusion criteria were the following: consensuses on the diagnosis of sarcopenia and studies developing and validating sarcopenia screening tools in older adults written in English or Spanish. On the other hand, studies focused on evaluating sarcopenia on specific diseases (i.e., diabetes, cancer, or cardiovascular diseases) were excluded.

As a result of the literature search, several scales were found; of these, we selected the most relevant according to their clinimetric properties and practical usefulness for the screening of sarcopenia. The most outstanding and the most studied in different populations and languages were SARC-F and SARC-F-Calf (13, 27). However, within their theoretical models, they include an item for falls (“How many times have you fallen in the past year?”), which is considered a geriatric syndrome and a negative outcome of sarcopenia; therefore, it must not be included in the theoretical model of sarcopenia (strength, muscle mass, and slow gait speed or low score in the Chair stand test). In addition, something that has characterized SARC-F is its low sensitivity in different studies and populations (13, 27–29). As shown in several studies, adding the calf circumference to the SARC-F improves its sensitivity (SARC-CalF) (15, 28, 29). Other scales like the Mini Sarcopenia Risk Assessment (MSRA) questionnaire (16) and SarSA-Mod (17) are focused on evaluating characteristics related to the risk of sarcopenia and not specifically to its presence.

#### 2.3.2 Second stage: contextualization of the theoretical model

A multidisciplinary team that included geriatricians, internists, rehabilitation physicians, nutritionists, and physiotherapists analyzed the selected literature to build the theoretical model of sarcopenia and design the preliminary components of the scale. The team concluded that the items related to the three dimensions of sarcopenia (muscle strength, muscle mass, and gait speed) on which the current consensuses agree (9–11, 16) should be included in the scale.

#### 2.3.3 Third stage: conformation of the scale

The first preliminary version of the scale included an item pool with 41 subjective items. Following the Delphi (30) method, the multidisciplinary team evaluated the face validity and the content validity of each one of the items.

In the first work session, 80% of the team agreed to eliminate 31 items due to insufficient face or content validity. The second preliminary subjective version included 10 items that were tested in a pilot group of 15 adults aged 50 years or older to assess the comprehension of the questions. In the second work session, the team discussed the comprehension of the questions, and it was concluded that the participants of the pilot study had good comprehension of the questions. Therefore, the team decided to include the 10 items in the three rounds of the FraDySMex cohort (2014, 2015, and 2019). In a third work session, the team concluded that the main problem of subjective scales was their low sensitivity versus international consensuses. This could be due to the comparison between subjective items and objective criteria. To improve the above in the new scale, the team agreed to include subjective items and affordable objective proxy tests to evaluate muscular strength and muscle mass. The chosen tests were the Chair stand test and the measurement of calf circumference, based on their predictive validity for different outcomes (15, 29, 31–33). The inclusion of calf circumference to SARC-F has demonstrated an improvement in the criterion validity (sensitivity, specificity, and AUC) (15, 29). The addition of the Chair stand test in the SARCO-GS was considered since the low muscular strength that this test is able to assess has been proposed by international consensuses as a part of sarcopenia (confirmed or severe) (10), and because it has proven to be an excellent proxy to evaluate the strength of leg muscles (quadriceps muscle group). The cut-off points for these measurements were: >15 seconds on the Chair stand test (10) and ≤33 cm and ≤34 cm of calf circumference for women and men, respectively (15).

##### 2.3.3.1 Optimization of the scale length

In a fourth work session, the team analyzed the inter-item correlation. If the correlation between items was rho ≥ 0.90, then the items were discarded. Five items were eliminated, resulting in the final version of the SARCO-GS seven-item [one subjective item of gait speed, two subjective items of muscle strength, two subjective items of muscle mass, the Chair stand test, and calf circumference (**Table 1**)].

Table 1SARCO-GS, Spanish and English versions.

**Table:** 

Spanish Version			
Dimensiones	Items	Categorías	Puntaje
**Velocidad de la marcha subjetiva**	1. Desde hace 3 meses ¿Ha notado que camina…	Nada lento (normal)	0
		Un poco lento	1
		Muy lento o incapaz	2
**Medición subjetiva de fuerza muscular**	*2.* ¿Cuánta fuerza tiene para cargar algo pesado de 4 kilogramos o más? Ejemplo: cargar una cubeta o barrica o garrafón de llenas de agua o cargar dos bolsas de mandado o supermercado	Mucha	0
		Poca	1
		Nada o incapaz	2
	3. ¿Cuánta dificultad tiene para subir un piso de escaleras?	Ninguna	0
		Poca	1
		Mucha	2
**Medición subjetiva de cantidad de masa muscular**	4. En los últimos 3 meses: ¿Ha notado que sus piernas y/o brazos han enflaquecido?	Nada	0
		Poco	1
		Mucho	2
	5. En los últimos 3 meses: ¿Ha notado que sus piernas y/o brazos están más flacos o delgados comparado con las personas de su misma edad?	Nada	0
		Poco	1
		Mucho	2
**Medición objetiva de fuerza muscular**	6. Prueba de levantarse de la silla 5 veces	≤ 15 segundos	0
		≥ 16 segundos	2
**Medición objetiva de cantidad de masa muscular**	7. Circunferencia de pantorrilla	Mujer: >33 Hombre: >34	0
		Mujer: ≤33 Hombre: ≤34	2
Sarcopenia = ≥ 3 puntos del puntaje total.

**Table:** 

English Version			
Dimensions	Items	Categories	Score
**Subjective gait speed**	In the past 3 months, you have noticed that you walk…	Not slowly at all (normal)	0
		A little slowly	1
		Very slowly or unable	2
**Subjective muscular** **Strength**	1. How able do you feel to carry a heavy object? (at least 4 kilograms or 9 pounds) Example: carrying a bucket, barrel, or jug full of water or carrying two supermarket bags	Very	0
		Little	1
		Not at all or unable	2
	How difficult is it for you to climb up a flight of stairs?	Not at all	0
		A little	1
		Very	2
**Subjective muscle mass**	In the last three months, have you noticed that your legs and/or arms have become thinner?	Not at all	0
		A little	1
		Much	2
	In the last 3 months: Have you noticed that your legs and/or arms are skinnier or thinner compared to people your same age?	Not at all	0
		A little	1
		Much	2
**Objective muscular** **strength**	Chair stand test (Stand up from a chair 5 times)	≤ 15 seconds	0
		≥ 16 seconds	2
**Objective muscle mass**	Calf circumference	Female: >33 Male: >34	0
		Female: ≤33 Male: ≤34	2
Sarcopenia = ≥ 3 points.			

##### 2.3.3.2 Translation–retranslation

Once the final version was established, we translated the SARCO-GS into English to encourage its use among non-Spanish-speaking populations, following a standardized procedure (translation and retranslation) to adapt scales (34) (**Table 1**).

### 2.4 Validation (validity and reliability)

The final version of the SARCO-GS seven-item was subjected to reliability and validity analyses. All the analyses except those of predictive validity were performed with data from the 2019 round of FraDySMex since the sample size (n=852) was greater than the size of other rounds.

#### 2.4.1 Criterion validity

##### 2.4.1.1 Cut-off point selection

To determine the cut-off points, the AUC was estimated using the SARCO-GS total score versus the EWGSOP2 consensus, and the cut-off point with better sensitivity, specificity, and AUC was chosen.

Once the cut-off point of SARCO-GS was determined, we analyzed the sensitivity, specificity, AUC, and likelihood ratios of SARCO-GS versus EWGSOP2, FNIH, AWGS, SARC-F, and SARC-CalF as reference standards.

Additionally, to strengthen the criterion validity, we compared the AUC between SARCO-GS, SARC-F, and SARC-CalF (screening scales) using as reference the EWGSOP2 (10), FNIH (11), and AWGS (9) consensuses in order to evaluate which scale had the better AUC.

#### 2.4.2 Construct validity

To test if the SARCO-GS had adequate construct validity (convergent validity with other measurements), Spearman’s and Pearson’s correlation coefficients were estimated between each item and the total score versus the total score of other measurements related to the construct. The remaining related measurements were quality of life, IADL, ADL, presence of depressive symptoms, comorbidity, nutritional status, gait speed, physical performance, hand grip strength, and phase angle.

#### 2.4.3 Predictive validity

To strengthen the validity, we assessed whether sarcopenia was associated with an increased risk of functional disability. To evaluate the functional dependency, the ability to perform instrumental activities that could be considered complex was evaluated with the Lawton Instrumental Activities of Daily Living Scale (20). We chose this tool considering that the study participants were non–institutionalized adults who had to attend the evaluation centers; therefore, they were expected to have more independence in daily basic activities. To assess the above, we considered the basal measurements of the participants in the study (2014–2015) and the follow-up measurement of functional disability in 2019. A Cox model was performed, adjusting by the following potential confounder variables: age, sex, BMI, education, marital status, comorbidity, and cognitive impairment.

#### 2.4.4 Consistency (reliability)

Spearman’s correlation coefficients between items (inter-item) and the total score (item-total) were estimated to assess internal reliability. It was considered sufficient correlation if the coefficient between each item and the total score was significant and higher than 0.30 (35). In addition, the Cronbach’s alpha was estimated.

### 2.5 Statistical analysis

In the descriptive analysis, means ± SD were used for continuous variables, as well as frequencies and percentages for categorical variables.

#### 2.5.1 Criterion validity

The cut-off point was determined with a frequency table and the AUC.

The sensibility, specificity, AUC, and likelihood ratios between SARCO-GS and EWGSOP2, FNIH, and AWGS were assessed through a frequency table and the AUC.

The AUC between SARCO-GS, SARC-F, and SARC-CalF using as reference the EWGSOP2, FNIH, and AWGS consensuses were graphed and compared to evaluate which scale had the better AUC.

#### 2.5.2 Construct validity

Spearman’s and Pearson’s (normal distribution) correlation coefficients between each item of the scale and the total score versus the total score of the other related measurements were estimated.

#### 2.5.3 Predictive validity

To evaluate if sarcopenia screening by SARCO-GS was an independent risk factor for functional dependence, the Hazard Ratios (HR) were estimated with a Cox regression model adjusting for other variables.

A p-value <0.05 was considered significant and we considered 95% confidence intervals (CI). All the analyses were conducted in STATA/SE 15.0.

## 3 Results

### 3.1 Sample characteristics

The characteristics of the study sample (FraDySMex round 2019) in which SARCO-GS was validated are in **Table 2**. The average age was 68.9 years (SD 10.21) and 80.1% were female, almost half (48.8%) were married or in a consensual union, and 71% were overweight or obese (40.9% and 30.4%, respectively). Regarding comorbidities, 77.5% had low comorbidity according to the Charlson Index.

**Table 2:** Baseline characteristics of the participants of FraDySMex cohort, Mexico City.

Age (years)	% (n)
50-69	56.2 (479)
≥70	43.8 (373)
Sex	
Female	80.1 (423)
Male	19.9 (105)
Marital status	
Married/consensual union	48.8 (415)
Single/divorced	26.12 (222)
Widower/widow	25.06 (213)
Body Mass Index (kg/m^2^)	
Normal (18.5-24.9)	28.0 (236)
Low weight (<18.5)	0.7 (6)
Overweight (25-29.9)	40.9 (345)
Obesity (≥30)	30.4 (256)
Comorbidity (Charlson Index)	
Low comorbidity (<3 points)	77.5 (606)
High comorbidity (≥3 points)	22.5 (192)
Sarcopenia (SARCO-GS)	
No sarcopenia (<3 points)	45.4 (383)
With sarcopenia (≥3 points)	54.6 (461)

### 3.2 Criterion validity

#### 3.2.1 Cut-off point selection

The final total score of SARCO-GS was set from 0 to 14 points. The selected cut-off point to screen sarcopenia with SARCO-GS was ≥3 from the total score (**Table 2**). This value had better sensitivity (77.68%), specificity (53.71%), and AUC (0.73) (considering the EWGSOP2 consensus as a reference) (**Table 3**).

**Table 3:** Different cut-off points of SARCO-GS and their sensibility, specificity, and likelihood ratios versus EWGSOP2.

Cut-off point	Sensitivity (%)	Specificity (%)	LR (+)	NLR (-)
≥0	100.00	0.00	1.00	–
≥1	93.30	24.03	1.23	0.28
≥2	88.55	40.12	1.48	0.32
≥3*	**77.68**	**53.71**	**1.68**	**0.42**
≥4	66.96	67.58	2.10	0.49
≥5	50.89	80.23	2.37	0.63
≥6	37.95	88.43	3.02	0.71
≥7	29.46	93.12	3.89	0.76
≥8	20.54	94.44	3.35	0.85
≥9	14.29	96.34	3.54	0.89
≥10	8.93	98.24	4.61	0.93
≥11	3.12	99.12	3.23	0.98
≥12	1.34	99.56	2.77	0.99
≥13	0.45	99.71	1.38	1.00
≥14	0.00	99.85	0.00	1.00

AUC = 0.73.

*Selected cut-off point to screen sarcopenia.Positive likelihood ratio (LR+), negative likelihood ratio (LR+), European Working Group on Sarcopenia in Older People (EWGSOP).

SARCO-GS had higher values of sensitivity and AUC than SARC-F and SARC-Calf using EWGSOP2 and FINH as references. Regarding specificity, SARC-F and SARC-Calf obtained higher values than SARCO-GS (**Table 4**).

**Table 4:** Criterion validity of SARCO-GS (≥ 3 points), SARC-F and SARC-CalF versus sarcopenia consensuses.

	Sensitivity (%)	Specificity (%)	LR (+)	NLR (-)	AUC
EWGSOP2					
SARCO-GS	77.68	53.71	1.68	0.4156	0.73
SARC-F	22.77	91.49	2.68	0.84	0.62
SARC-CalF	37.95	85.07	2.54	0.73	0.69
FNIH					
SARCO-GS	74.15	52.44	1.56	0.49	0.67
SARC-F	23.90	92.39	3.14	0.82	0.62
SARC-CalF	31.22	83.50	1.89	0.82	0.62
AWGS					
SARCO-GS	77.57	48.71	1.52	0.46	0.70
SARC-F	17.76	88.51	1.54	0.93	0.56
SARC-CalF	50.47	83.24	3.012	0.59	0.74

European Working Group on Sarcopenia in Older People (EWGSOP), Foundation for the National Institutes of Health (FNIH), Asian Working Group for Sarcopenia (AWGS 2019), positive likelihood ratio (LR+); negative likelihood ratio (LR-).

**Figure 1:**
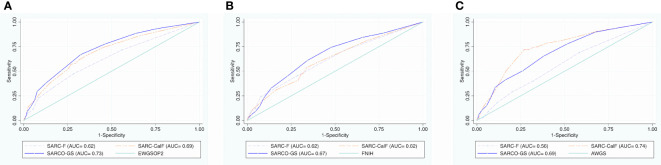
Comparative AUC between SARCO-GS, SARC-F, and SARC-CalF versus EWGSOP2, FNIH, and AWGS criteria. **(A)** Comparative AUC between SARCO-GS, SARC-F, and SARC-CalF versus EWGSOP2 criteria. **(B)** Comparative AUC between SARCO-GS, SARC-F, and SARC-CalF versus FNIH criteria. **(C)** Comparative AUC between SARCO-GS, SARC-F, and SARC-CalF versus AWGS criteria.

**Figures 1A-C** show the comparative AUC between SARCO-GS, SARC-F, and SARC-CalF, considering EWGSOP2 (**Figure 1A**), FNIH (**Figure 1B**), and AWGS (**Figure 1C**) as references. SARCO-GS had a higher AUC than SARC-F and SARC-CalF when the EWGSOP2 and the FNIH were considered as references. However, when AWGS was used as a reference, SARCO-GS had a better AUC than SARC-F but not better than SARC-CalF.

### 3.3 Construct validity

The SARCO-GS had adequate construct validity (convergent) since there was a significant correlation between the dimension that evaluates each item, the total score, and other objective/subjective measurements (**Table 5**). Item 1 (subjective gait speed) was correlated with IADL and ADL, Charlson Index, depression symptoms, gait speed, hand grip strength, and physical performance; items 2 and 3 (subjective muscular strength) were correlated with quality of life, IADL and ADL, depression symptoms, nutritional status, gait speed, hand grip strength, and physical performance; both items 4 and 5 (subjective muscular mass) were correlated with nutritional status and item 4 was also correlated with depression symptoms, physical performance, and angle phase. The results of the Chair stand test were correlated with IADL and ADL, nutritional status, hand grip strength, physical performance, and angle phase, whereas calf circumference only correlated with angle phase. All the assessed measurements were correlated with the SARCO-GS total score.

**Table 5:** Construct validity of SARCO-GS (convergent and divergent) by correlations with other measurements.

Variable	Item 1		Item 2		Item 3		Item 4		Item 5		Item 6		Item 7		Total score	
Total score	rho	P	rho	p	rho	p	rho	p	rho	p	rho	p	rho	p	rho	p
Quality of life EQ-VAS	-0.14	<0.001	-0.20	<0.001	-0.25	<0.001	-0.15	0.001	-0.12	<0.001	-0.15	0.002	-0.02	0.5487	-0.22	<0.001
ADL Barthel Index	-0.28	<0.001	-0.30	<0.001	-0.34	<0.001	-0.18	<0.001	-0.09	0.007	-0.27	<0.001	-0.10	0.002	-0.33	<0.001
IADL Lawton scale	-0.29	<0.001	-0.40	<0.001	-0.36	<0.001	-0.14	<0.001	-0.10	0.002	-0.37	<0.001	-0.19	<0.001	-0.41	<0.001
Depression symptoms CESD-7	0.29	<0.001	0.28	<0.001	0.29	<0.001	0.23	<0.001	0.19	<0.001	0.19	<0.001	0.04	0.238	0.34	<0.001
Comorbidities Charlson Index	0.24	<0.001	0.20	<0.001	0.20	<0.001	0.13	<0.001	0.09	0.012	0.17	<0.001	-0.006	0.856	0.24	<0.001
Nutritional status MNA	-0.35	<0.001	-0.31	<0.001	-0.32	<0.001	-0.32	<0.001	-0.30	<0.001	-0.23	<0.001	-0.17	<0.001	-0.45	<0.001
Gait speed (m/seg)	-0.25	<0.001	-0.25	<0.001	-0.23	<0.001	-0.18	<0.001	-0.13	<0.001	-0.09	0.013	-0.15	<0.001	-0.36	<0.001
Grip strength (kg)	-0.28	<0.001	-0.37	<0.001	-0.33	<0.001	-0.18	<0.001	-0.11	0.002	-0.27	<0.001	-0.19	<0.001	-0.36	<0.001
Physical performance SPPB	-0.39	<0.001	-0.41	<0.001	-0.40	<0.001	-0.23	<0.001	-0.19	<0.001	-0.64	<0.001	-0.13	<0.001	-0.57	<0.001
Angle phase (°)	-0.18	<0.001	-0.19	<0.001	-0.18	<0.001	-0.21	<0.001	-0.16	<0.001	-0.35	<0.001	-0.22	<0.001	-0.36	<0.001

EuroQol visual analog scale (EQ-VAS), basic activities of daily living (ADL), instrumental activities of daily living (IADL), Center for Epidemiologic Studies Depression Scale Short Form (CESD-7), Mini Nutritional Assessment (MNA), Short Physical Performance Battery (SPPB).

### 3.4 Predictive validity

Sarcopenia screened by SARCO-GS increased the risk of functional dependence (HR: 2.33, CI 95% 1.02-4.88, p-value=0.046) in adults aged 50 years or older in 4.2 years (average) of follow-up (**Table 6**). Therefore, SARCO-GS had predictive validity concerning functional dependence, which is an adverse outcome of sarcopenia.

**Table 6:** Predictive validity of SARCO-GS: Adjusted Hazard ratios for functional dependence.

	Hazard ratio	p-value	CI 95%
Sarco-GS			
No sarcopenia (<3 points)	1.00		
With sarcopenia (≥3 points)	2.22	0.046	1.01-4.88
Sex			
Female	1.00		
Male	0.61	0.238	0.27-1.39
Age (years)			
50-69	1.00		
≥70	2.31	0.014	1.19-4.49
Body Mass Index (kg/m^2^)			
Normal (18.5-24.9)	1.00		
Low weight (<18.5)	0.81	0.533	0.43-1.55
Overweight (25-29.9)	0.59	0.148	0.29-1.21
Obesity (≥30)			
Marital Status			
Married/consensual union	1.00		
Single/divorced	1.12	0.741	0.56-2.26
Widow/widower	0.88	0.648	0.46-1.66
Education (years)			
≥13	1.00		
7-12	1.43	0.389	0.63-3.26
<7	1.81	0.199	0.73-4.49
Comorbidity (Charlson Index)			
Low comorbidity (<3 points)	1.00		
High comorbidity (≥3 points)	1.06	0.859	0.58-1.92
Cognitive impairment (MMSE)			
No	1.00		
Yes	0.84	0.679	0.37-1.91

MMSE, Mini-Mental State Examination (score ≤23 in the case when the years of study were ≥5; score ≤19 if the years of study were between 1 and 4; score ≤ 16 if the years of study were <1).

### 3.5 Consistency (Reliability)

**Table 7** shows the internal reliability of SARCO-GS. The Spearman’s correlation coefficients between each item and the total score were in a range from 0.50 to 0.70 (moderate and good correlations). The Cronbach’s alpha was 0.67, which is close to 0.70, an acceptable value for reliability (36).

**Table 7:** Internal Reliability of SARCO-GS, by correlation inter-item, item total, and Cronbach’s alpha (rho, p-value).

Domain	Item	1	2	3	4	5	6	7	Total score
**Subjective gait speed**	**1**	1.00							0.59 <0.001
**Subjective muscular strength**	**2**	0.43 <0.001	1.00						0.62 <0.001
**Subjective muscular strength**	**3**	0.45 <0.001	0.53 <0.001	1.00					0.59 <0.001
**Subjective muscle mass**	**4**	0.22 <0.001	0.23 <0.001	0.18 <0.001	1.00				0.57 <0.001
**Subjective muscle mass**	**5**	0.20 <0.001	0.24 <0.001	0.21 <0.001	0.58 <0.001	1.00			0.54 <0.001
**Muscular strength**	**6**	0.30 <0.001	0.32 <0.001	0.35 <0.001	0.19 <0.001	0.16 <0.001	1.00		0.62 <0.001
**Muscle mass**	**7**	0.06 0.031	0.11 0.001	0.07 0.057	0.21 <0.001	0.22 <0.001	0.13 <0.001	1.00	0.51 <0.001

Cronbach Alpha: 0.67.

## 4 Discussion

The structure of the new SARCO-GS (subjective items on strength, muscle mass, and gait speed plus the Chair stand test and calf circumference) proposes an innovative manner to screen the sarcopenia construct based on the recommendations of international consensuses on sarcopenia (9–11). This mixed composite structure (subjective and objective) was built based on current evidence that has reported that the inclusion of objective items ameliorates the low sensitivity showed by totally subjective scales such as SARC-F (this low sensitivity is observed when subjective scales are compared with objective diagnosis criteria). In our study, the sensitivity demonstrated by SARCO-GS versus EWGSOP2, FNIH, and AWGS was good. SARCO-GS demonstrated a higher sensibility than SARC-F and SARC-CalF versus EWGSOP2, FNIH, and AWGS. The low sensitivity of SARC-F observed in our study is consistent with that reported in multiple studies (13, 27–29) and summarized in a systematic review and meta-analysis (14) in which values from 28.9% to 55.3% were reported versus EWGSOP2, FNIH, and AWGS. Similarly, SARC-CalF sensitivity has obtained a range from 15.7% to 60.7% versus the mentioned consensuses in other studies (28, 29, 37, 38). Regarding the specificity, SARC-F and SARC-CalF obtained higher values than SARCO-GS versus the three consensuses used as references. However, in a scale intended for population screening, a higher sensitivity is more desirable than specificity due to the importance of decreasing the number of false negatives (14, 39). In other studies, it has been observed that SARC-F has higher values of specificity than of sensibility (range value from 15% to 96.5% versus EWGSOP2; 79.3% to 99.2% versus FNIH; 15.1% to 98.4% versus AWGS); this is also the case for SARC-CalF (29, 38, 40, 41).

Another property of SARCO-GS is that its AUC reflected a good quality when EWGSOP2 was used as a reference. This value was higher than the one obtained by SARC-F (0.62). In other studies (14, 29), a wide range of AUC values has been observed for SARC-F versus EGWSOP from 0.51 to 0.87. Also, SARCO-GS had a higher AUC (0.67) than SARC-F (0.62) versus FNIH. In other studies (14, 29, 37, 40), SARC-F obtained AUC values from 0.68 to 0.89. Regarding AWGS, the AUC of SARCO-GS (0.70) was higher than that of SARC-F (0.56); the AUC of SARC-F reported in this study was inside the range reported in other studies (0.53 to 0.92) (14, 29, 37). On the other hand, the AUC of SARCO-GS was also higher than SARC-CalF (0.69) versus EWGSOP and FNIH; other studies (29, 37, 38, 40) have reported that this value ranges from 0.59 to 0.85 using the EWGSOP2 as a reference and from 0.68 to 0.89 using the FNIH consensus. SARC-CalF had a higher value of AUC (0.74) than the value observed for SARCO-GS versus AWGS. In other populations (29, 38), the AUC range for the SARC-CalF versus AWGS was between 0.73 and 0.92. The above could be explained by the high specificity of SARC-CalF.

The variability in the AUC values of SARC-F and SARC-CalF could be explained by the differences in the prevalence of sarcopenia, the adjusted cut-off points, and the specific characteristics of each studied population.

Taking into account the results obtained in the evaluation of the criterion validity, SARCO-GS had a better ability to detect sarcopenia cases than SARC-F and SARC-CalF using as references EWGSOP2 and FNIH.

Additionally, the results obtained from the construct validity assessment verified that all SARCO-GS items (subjective and objective) and the total score are correlated with the proxy objective constructs included in the FraDySMex cohort. For example, the gait speed item (Item 1) was correlated with gait speed as measured by the GAITRite, which has proven to be a gold standard for gait speed assessment (41). The muscle strength items (2 and 3) that assessed the strength to carry a heavy object (upper extremity, item 2) and to climb stairs (lower extremity, item 3) were correlated as expected with hand grip strength and with the SPPB; both tests have been considered in the EWGSOP2 as proxy assessments of arm and leg muscle strength (10). The items of subjective perception of muscle mass (items 4 and 5) were correlated with the phase angle, which is a proxy measurement for assessing muscle mass and has been associated with frailty and sarcopenia (42). Moreover, these items were also correlated with the MNA, which evaluates the risk of malnutrition, which is an indicator related to muscle mass quantity (43).

The Chair stand test was correlated with hand grip strength and phase angle. The above had concordance with the existing evidence; this test has been associated with muscular strength (44) and muscle mass (32). The calf circumference had a significant correlation with the phase angle that reflected the quantity of muscle mass (42) and MNA. Since these items are quantifiable, their inclusion in SARCO-GS improves the capacity of the scale to identify individuals affected by sarcopenia. Other constructs such as quality of life, depression, disability, and comorbidity were correlated with the SARCO-GS total score; these findings agree with the evidence on the association between these constructs and sarcopenia (27).

Regarding predictive validity, functional disability is one of the main adverse outcomes of sarcopenia (45, 46) and our results using the SARCO-GS are congruent with these findings. Sarcopenia evaluated by SARCO-GS increased the risk of functional disability in a follow-up period of 4.2 years. Therefore, the proposed cut-off point ≥3 is useful for intervention and longitudinal studies to prevent this outcome. These results strengthen the criterion validity of SARCO-GS.

This predictive capacity of SARCO-GS confirms that the inclusion of calf circumference and Chair stand test strengthens the construct of sarcopenia (10) and provides support to be included in the screening stage.

The internal reliability by inter-item and item-total was acceptable. Even though this scale is composed of objective and subjective measurements, it shows that all the items belong to this same construct of sarcopenia. The Cronbach alpha of 0.69 was reasonable; although this coefficient is helpful for comparative purposes between other populations, it belongs to classical theories of psychometry and has the disadvantage that, when it is employed to analyze the internal structure of scales that combine subjective and objective clinical evaluations with different variances, the value may be low. In these cases, its interpretation should be considered carefully (47).

It is important to consider some limitations of the present study. The studied population in which SARCO-GS was validated is a representative sample of three districts in the southeast of Mexico City; therefore, it considers unique characteristics of this population. Another limitation is the lack of evaluation of the external reliability (test re-test or inter-rater agreement). Considering these limitations, it is crucial to validate SARCO-GS in populations other than Mexico and worldwide. Some strengths should be mentioned: the present study comprised data from a longitudinal cohort study that included the measurements assessed by objective tools like DXA, hand dynamometer, and GAITRite.

## 5 Conclusions

SARCO-GS is a new scale to screen sarcopenia that combines subjective items with objective measurements. The SARCO-GS yielded satisfactory results in terms of sensitivity, AUC versus the most used consensuses, predictive validity for functional disability, construct validity, and internal reliability. SARCO-GS could narrow the gap of subjective scales in terms of sensitivity to timely screening of sarcopenia in community-dwelling adults and prevent adverse outcomes. Furthermore, it could be used in different clinical and research settings since its measurements do not require specialized equipment and are easy to conduct.

## Data availability statement

The raw data supporting the conclusions of this article will be made available by the authors, without undue reservation.

## Ethics statement

The studies involving human participants were reviewed and approved by National Institute of Geriatrics (DI-PI-002/2014). The patients/participants provided their written informed consent to participate in this study.

## Author contributions

Conceptualization: OR-C. Methodology: OR-C, IO-G, AIG-G, and AL-L. Data analysis: IO-G and OR-C. Writing—Original Draft Preparation: OR-C and IO-G. Writing—Review and Editing: OR-C, IO-G, AIG-G, and AL-L. All authors contributed to the article and approved the submitted version.
